# For sustainability environment: some determinants of greenhouse gas emissions from the agricultural sector in EU-27 countries

**DOI:** 10.1007/s11356-024-33273-2

**Published:** 2024-04-23

**Authors:** Hasan Gökhan Doğan, Mustafa Kan

**Affiliations:** Agricultural Faculty, Department of Agricultural Economics, Kırşehir Ahi Evran University, 40100 Kırşehir, Kırşehir Province Türkiye

**Keywords:** Climate change, Global warming, Produce residues, Enteric fermentation, EU-27, Panel data

## Abstract

Climate events significantly affect the lives of not only humanity but also all living things. Just as transformation in the ecosystem affects sectors, all sectors also transform the ecosystem. It is stated that the agricultural sector is at the root of the deterioration in the ecosystem due to the effect of intensive agriculture after the green revolution. It can be stated that, with an understanding far from the concept of sustainability, the foodstuffs and their waste produced in the agricultural sector are considered among the causes of climate change, which is now concentrated on the whole world in the third millennium. In this study, the effect of N_2_O gas released from produce residues and the release of enteric fermentation on the level of CO_2_ released from agricultural-food systems was investigated using advanced econometric models. The findings reveal that both factors are effective. However, it can be stated that the effect of N_2_O gas released from the produce residues is greater. Suggestions such as improving feed rations and maintaining herd management strategies within certain patterns to reduce the level of enteric fermentation may contribute to the process. In produce residue management, turning waste into compost and expanding bioenergy power plants will ensure both waste disposal and resource continuity in generating energy. Otherwise, the decreasing resources in the world may come to an end, and there will be disruptions and problems in the agricultural sector, as in all sectors. Considering the increasing world population, it is inevitable that food supply security may be endangered and the hunger problem may reach an irreversible level.

## Introductıon

**Fig. 1:**
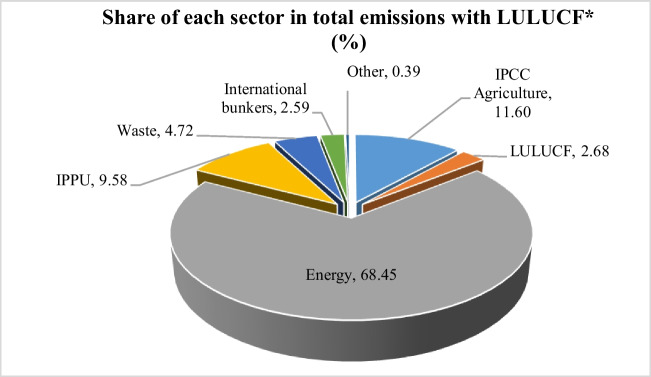
Share of sectors greenhouse gas emissions (%) (FAO 2023a, b)

Climate change is one of the most serious problems that threaten the future of humankind. In a study conducted on global risks, it is stated that the top three most important risks in the long term are failure to achieve success in the fight against climate change and the resulting natural disasters (World Economic Forum (WEF) 2023). Greenhouse gasses in the atmosphere, which are shown to be one of the main causes of climate change, started to increase after the Industrial Revolution that started in the 1750s, and the Intergovernmental Panel on Climate Change (IPCC) states that these increases are definitely caused by human activities (IPCC 2013). In addition to occurring naturally, greenhouse gasses occur because of various human activities (Köknaroglu and Akünal 2010). Energy is a sector that plays the largest role in greenhouse gas emissions (Fig. 1).

Agriculture, a sector that significantly affects greenhouse gas emissions, is a sector that is affected by climate change. Within the scope of the UN Sustainable Development Goals, “SDG 13: Climate action” is directly related to agricultural production (United Nations 2023). Sustainable land use, the future of food, the future of consumption, etc. Within the framework of the discussions, it is recommended that new agricultural practices and technologies be developed in the fight against climate change. On the other hand, the development of agricultural practices that contribute to the reduction of GHG emissions will contribute to the mitigation side of the fight against climate change (Kara and Yereli 2022).

**Fig. 2:**
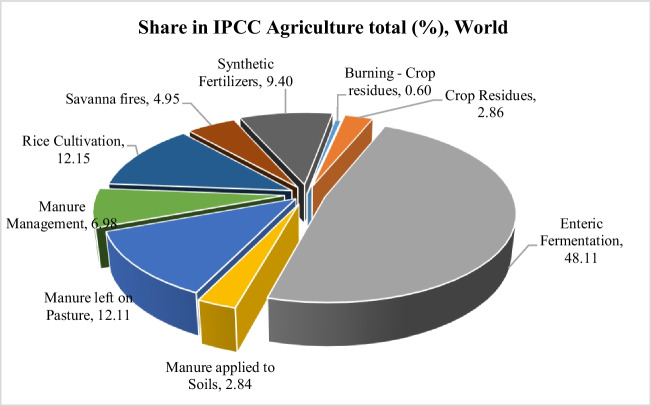
Share of agricultural activities in climate change (%) (FAO 2023a, b)

Considering that approximately 2.5 billion people in developing countries earn their living from agriculture, it is clear to what extent climate change will threaten human welfare and agricultural production. Agriculture is a basic and strategic sector known for its contribution to the nutrition of people all over the world, national income and employment, providing raw materials and capital to the industrial sector, and its contributions to biodiversity and ecological balance (Doğan et al. 2015). In parallel with the ever-increasing world population, the need for food is increasing day by day. Greenhouse gasses such as CO_2_ (carbon dioxide), CH_4_ (methane), and N_2_O (nitrous oxide), which occur as a result of agricultural activities (energy consumption, plant and animal production, fertilization, pesticide use, etc.), are considered among the causes of climate change (Akalın 2014). Important agricultural activities that affect greenhouse gas emissions are presented in Fig. 2.

Among the effects of agricultural activities on climate change, main activities such as energy consumption, production, animal husbandry, fertilization, and spraying, as well as N_2_O release resulting from waste in plant production and CH_4_ release resulting from enteric fermentation because of livestock activities, are also important factors (Fig. 2). When the figures for 2021 are examined, it is stated that the most important contributors to greenhouse gas emissions are CO_2_ emissions from deforestation and methane (CH_4_) emissions from the enteric fermentation of ruminant livestock (2.9 gigaton CO_2_eq each). This represents 40% of the total agri-food system. Other important components are CH_4_ emissions from animal manure (manure management, soil application, and manure deposition) and agri-food system waste disposal, each around 1.3 Gt CO_2_eq (FAO 2023a, b). Among the greenhouse gasses, N_2_O has a 265 times stronger effect on global warming than CO_2_ (IPCC 2013). In the last 200 years, atmospheric concentrations of important greenhouse gasses, CO_2_, CH_4_, and N_2_O, have increased significantly. El-Fadel and Massoud (2001) stated that the increase in greenhouse is caused by the production and use of fossil fuels, agricultural activities, and industrial activities. According to the (IPCC 2007a, b), the global warming potential values of CO_2_, CH_4_, and N_2_O gasses over a 100-year period are reported to be 1, 21, and 310 carbon dioxide equivalents, respectively. Greenhouse gasses such as CH_4_ and N_2_O, which are produced by waste and enteric fermentation, are gaining importance as gasses that contribute significantly to global warming because of their high global warming potential. Given that these gasses originate from enteric fermentation and agri-food systems, there are many proposals to reduce emissions. However, reducing production in the face of increasing food demand will be a major challenge. However, without a reduction in production, it is better to manage wastes and emissions properly and to create policies to do so. This is what this research focuses on.

This study aims to evaluate the impact of produce residues and greenhouse gas emissions resulting from enteric fermentation on the emissions released within the scope of the agricultural food system in the EU 27 countries.

## Materıal and method

The symbols, units, and data sources regarding the share of emissions released from agriculture-food systems in total emissions, the amount of N_2_O released from produce residues, and the amount of CO_2_ emissions resulting from enteric fermentation between 2000 and 2020 for the EU-27 countries are given in Table 1. In the third millennium, while developing policies to respond to the nutritional opportunities of the growing population, policies have also been sought in terms of environmental pressure and cost. Therefore, post-2000 research is gaining importance. In these policies, the EU is decisive. Because many economically developed countries of the world are located in this region. Since it is a regional structure, it is an important formation both agriculturally and commercially.

**Table 1:** Variables, symbols, and data sources used in the study

Variables	Symbol	Unit	Data source
Share of emissions released from agricultural– food systems emissions (CO_2_eq)	*⅄*	%	FAOSTAT
N_2_O released from the produce residues	*₱*	kt	FAOSTAT
Greenhouse gas emissions from enteric fermentation (CO_2_eq)	*ℇ*	kt	FAOSTAT

For the EU-27 countries determined as the research area, a panel data set was used using the variables given in Table 1. After the dataset was created, the “dog-log” model in semi-logarithmic form was preferred to provide interpretation opportunities because the unit of the dependent variable was proportional. Equation e = ꞵit/Y was used to determine the elasticities of the coefficients obtained in the dog-log (semi logarithmic function) models. Because, logarithmic functions allow exponential functions to be linearized and interpreted as percentages. However, in our equation, the dependent variable is already included in the model as a percentage. The functional relationship between the variables can be expressed as in Eq. 1.


1


After the hypothetically put forward functional relationship, the following panel data analyses were carried out to determine the impact levels of the emissions released from agricultural-food systems on the share of total emissions:

(i) Panel unit root test
(ii) Panel Toda–Yamamato causality test
(iii) Panel ARDL test

### Panel unit root test

In panel data econometrics, both time and differences between units can be examined together (Cameron and Trivedi 2005). Panel data creates a dataset consisting of *t* time and *k* variables for *n* cross-sections, thus allowing time and group effects to be included in the model. As in time series analysis, whether variables contain unit roots in panel data models should be investigated. Engle and Granger (1987) demonstrated that if the series are not stationary at their levels, the coefficients obtained from classical regression will be invalid. When working with a non-stationary series, the long-term relationship between the series disappears, and the relationship between the series does not reflect the truth. Many unit root tests are used in practice to investigate the stationarity of a series. The first-generation tests developed by Maddala and Wu (1999), Levin et al. (2002), Hadri (2000), Choi (2001), and Im et al. (2003) can be shown as an example of unit root tests (Doğan 2018).

In this study, panel unit root tests introduced by Levin et al. (2002) and Im et al. (2003) were preferred. Unlike the others, this test considers the heterogeneous structure in the panel data set as it first tests whether there is a unit root for each horizontal section separately to obtain panel-specific results (Güloğlu and İspir 2009).

2 $$\Delta {Y}_{it}={\alpha }_{i}{Y}_{it-1}+{\sum }_{j=1}^{p}{\beta }_{ij}\Delta {Y}_{it-j}+{X}_{it}{\delta }_{i}+{\varepsilon }_{it}$$

where *X* represents the constant and/or deterministic trend variables.

### Panel ARDL test

The ARDL bounds test approach allows co-integration testing with series that are not stationary at the same level (Pesaran and Shin 1995; Pesaran et al. 2001). The advantage of the ARDL approach is that co-integration testing can be performed without considering the degree of integration of the variables. In this study, the pooled mean group (PMG) estimator developed by Pesaran et al. (1999) was used to estimate long-term coefficients. The PMG estimator allows constant error term variances and short-run coefficients to vary. On the other hand, it allows the assumption of heterogeneity for short-term coefficients and homogeneity for long-term coefficients. The model created by following the ARDL approach developed by Pesaran et al. (1999) and using the PMG estimator is given in Eqs. 3, 4, and 5.


3



4



5


In Eqs. 3,4,5, *Δ* refers to the difference processor, and *m* refers to the lag length. Information criteria such as AIC, SC, FPE, and HQ are used to determine the lag length. Here, the lag length that provides the smallest critical value is determined as the lag length of the model (Doğan 2018).

### Panel Toda–Yamamato causality test

In this study, the Wald test (MWALD) recommended by Toda and Yamamoto (1995) was used. The Toda–Yamamoto Test ignores the I(0) and I(1) incompatibility between the series and the assumption of co-integration (Zapata and Rambaldi 1997; Wolde-Rufael 2004, 2005). This eliminates the difficulties and nativities that may arise in other causality tests. The MWALD test minimizes the risks related to the co-integration orders of the series by creating a VAR model according to the levels of the variables (Mavrotas and Kelly 2001). In this process, the appropriate “k” lag is determined, the maximum stationarity level dmax is determined, and the VAR model is solved with the lag *k* + dmax (Rambaldi 1997; Zapata and Rambaldi 1997). The results obtained have an asymptotic distribution and are not biased. Notations for the Toda–Yamamoto causality test are given in Eqs. 6, 7, and 8. The causality relationship and direction were determined by analyzing the following VAR system adapted for this study.


6



7



8


Models are analyzed by applying the “Seemingly Unrelated Regression” (SUR) procedure.

## Empirıcal results

Descriptive statistics for the variables included in the study are given in Table 2.

**Table 2:** Descriptive statistics for variables included in the study

	*⅄*	*ℇ*	*₱*
Mean	45.62	216.95	2.16
Median	29.18	96.79	0.89
Maximum	1593.68	1471.06	15.49
Minimum	− 19.95	1.16	0.0004
Std. Dev	105.17	309.03	3.14
Skewness	10.99	2.33	2.42
Kurtosis	142.52	8.18	8.71
Sum	25,869.50	123,014.93	1226.11
Sum Sq. Dev	6,260,891.74	54,056,076.96	5583.44

LLC and IPS unit root test results for the *⅄*, *₱*, and *ℇ* variables used in this study are given in Table 3.

**Table 3:** Panel unit root test results

Unit root test method	I (0)			I (1)		
	*⅄*	*₱*	*ℇ*	***∆**** ⅄*	***∆**** ₱*	***∆**** ℇ*
Levin, Lin and Chu *t* stat	0.14	− 5.57*	− 3.29*	− 6.41*	− 9.07*	− 3.95*
Im, Pesaran, and Shin *W*-stat	− 0.63	− 5,86*	− 1.25	− 8.04*	− 13.74*	− 5.55*

*Significant at 1% level

When the LLC and IPS unit root test results for the variables are examined, it can be said that *⅄* contains a unit root at I(0) and is stationary at the I(1) level compared with LLC and IPS. *₱* is stationary at both I(0) and (1) levels according to LLC and IPS, and *ℇ* has a unit root at I(0) level according to LLC and I(1) level according to IPS. It can be stated that it contains but is stationary at the I(1) level.

The results of the Toda–Yamamato causality test, which was constructed with VAR (vector auto regression) systems and considered the *k* + dmax lag length and stationarity level, are given in Table 4. Causality was analyzed in one way. Since the main purpose of this study is to reveal the severity of the parameters affecting the share of CO_2_ released from agricultural food systems, one-way analysis results are expressed.

**Table 4:** Panel Toda–Yamamato causality test results

Model	*k* + dmax	Wald test	Causality
₱-⅄	11	26.79*	₱⅄
ℇ-⅄	11	20.92**	ℇ⅄

* and ** indicate significance at 1% and 5% levels, respectively

When the results of the Toda-Yamamato causality test were examined, causality was determined from the amount of N_2_O released from produce residues to the share of the agricultural-food systems in total emissions and from enteric fermentation to the share of the CO_2_ level released from the agricultural-food systems. The severity of this relationship is expressed by the panel ARDL results in Table 5.

**Table 5:** Long-term and short-term panel ARDL model results

Dependent variable: ⅄		
Method: ARDL		
Independent variables: ₱, ℇ		
Selected model: ARDL(1,2,2)		
Variable	Coef	Elasticity
Long-run coefficient		
*₱*	2.17*	0.05
*ℇ*	0.62***	0.01
Constant	25.56*	
Short-run coefficient		
Cointeq	− 0.07**	− 0.001
*₱*	7.63**	0.016
*₱*-1	5.64**	0.12
*ℇ*	31.41	0.69
*ℇ*-1	− 51.72	− 1.13

*, **, *** indicate significance at 1%, 5%, and 10% levels, respectively. as trend specification was preferred rest.cons in the research and the maximum lag length was determined as 1

After determining whether the variables were affected by their previous values with the unit root test, the ARDL test was performed to reveal their long-term relationships. The ARDL test allows investigating the relationship of co-integration in the long run, regardless of whether the variables examined are stationary at the same level. ARDL long-term and short-term test results are given in Table 5.

When the results of ARDL (1,2,2) are examined, both variables examined on the share of CO_2_ emissions from agricultural-food systems in total emissions are statistically significant in the long term. When the amount of N_2_O released from produce residues increases by 1%, the share of CO_2_ released from agricultural food systems increases by 0.05%. Conversely, when the amount of CO_2_ resulting from enteric fermentation increases by 1%, the share of CO_2_ released from agricultural food systems increases by 0.01%. In the short term, it can be said that the effect of the N_2_O level released from the residues in the current period and the *t*–1 period is statistically significant. When the sources of agricultural CO_2_ emissions are examined, it can be stated that the N_2_O level released from produce residues and the CO_2_ level resulting from enteric fermentation are essential (FAO 2023a, b).

## Recommandatıon and conclusıon

Today, when climate change is felt more and more each day, countries are making commitments to reduce greenhouse gas emissions and develop policies and strategies on this issue (Bulut et al. 2023). The European Union, the world’s largest economic and political union, creates significant impacts all over the world with the policies it implements. One of these is the agricultural sector. It is known that 60% of the methane released from anthropogenic sources is released from agricultural activities, whereas 25% is released from enteric fermentation (Singh and Singh 2012; Keser and Kutay 2021). EU countries, which try to direct agriculture and rural areas through the Common Agricultural Policy, are taking important steps toward becoming a carbon–neutral and environmentally friendly community in the future. The effect of emissions resulting from produce residues and enteric fermentation on the greenhouse gas emissions released from the agricultural food system was found to be statistically significant in the short and long term. The findings obtained also coincide with reality. It can be said that livestock activities are essential in reducing greenhouse gas emissions caused by the agricultural sector. There is an intense release from both enteric fermentation and animal fertilizers. However, enteric fermentation has a greater impact. Various strategies can be put forward to combat this situation. First feeding is essential in animal husbandry activities. This is a paradox where, on the one hand, there is the efficiency and quality to be achieved per unit animal, and on the other hand, there are measures to be taken against the environmental costs. However, it is not insoluble. There are studies that recommend the use of various additives (yeast, probiotic, organic acid, etc.) to better utilize the energy of the feed and reveal their methane-reducing effect (Altan and Acar 2022). As the carbohydrate and fat content in the diet increases, methane emissions can be reduced (Moss et al. 2000; Meral and Biricik 2013). Additionally, as the amount of roughage increases in the ration of animals consuming high amounts of dry matter, methane emissions also increase (McAllister et al. 2011). On the other hand, within herd management strategies, improving genetic materials and removing unproductive animals from herds can have a positive effect on ensuring optimum efficiency. This also can reduce methane (Eckard et al. 2010; Pragna et al. 2018). In countries where livestock activities are intensive, continuing such practices with established policies may be beneficial in terms of adaptation and reduction. Produce residue management is another important issue. GHG emissions released from produce residues need to be managed. Otherwise, it may lead to the deterioration of the chemical structure of the atmosphere at both local and regional levels (Bencs et al. 2008). Although burning waste is prohibited in many countries, it continues to be burned for various reasons (removal of weeds, field preparation for the next year, disease control, etc.) (Gadde et al. 2009). The monitoring and evaluation processes of waste management must be within the framework of legal legislation. Political sanctions should be applied. Turning waste into compost and using it for plant nutrition will not only be beneficial in reducing emissions but also contribute to reducing the gasses that can be released by reducing the use of chemical fertilizers. On the other hand, producers’ plants produce residues that can be used in biomass power plants to obtain low-emission energy. Although there are difficulties such as transportation and storage, they can be traded within the scope of the clean development mechanism. For sustainable agricultural production and a sustainable environment, the interaction between waste obtained from agricultural producers and energy power plant investors may be a way out.
